# Use of a Network-Based Method to Identify Latent Genes Associated with Hearing Loss in Children

**DOI:** 10.3389/fcell.2021.783500

**Published:** 2021-11-29

**Authors:** Feng Liang, Xin Fu, ShiJian Ding, Lin Li

**Affiliations:** ^1^ Anaesthesia Department, China-Japan Union Hospital, JiLin University, Changchun, China; ^2^ School of Life Sciences, Shanghai University, Shanghai, China; ^3^ Department of Otorhinolaryngology Head and Neck Surgery, China-Japan Union Hospital of Jilin University, Changchun, China

**Keywords:** hearing loss, children, random walk with restart, protein-protein interaction, biomarker

## Abstract

Hearing loss is a total or partial inability to hear. Approximately 5% of people worldwide experience this condition. Hearing capacity is closely related to language, social, and basic emotional development; hearing loss is particularly serious in children. The pathogenesis of childhood hearing loss remains poorly understood. Here, we sought to identify new genes potentially associated with two types of hearing loss in children: congenital deafness and otitis media. We used a network-based method incorporating a random walk with restart algorithm, as well as a protein-protein interaction framework, to identify genes potentially associated with either pathogenesis. A following screening procedure was performed and 18 and 87 genes were identified, which potentially involved in the development of congenital deafness or otitis media, respectively. These findings provide novel biomarkers for clinical screening of childhood deafness; they contribute to a genetic understanding of the pathogenetic mechanisms involved.

## Introduction

Deafness refers to a total or partial inability to hear, also known as hearing impairment or hearing loss (Olusanya et al., 2019). According to the World Health Organization, approximately 5% of people worldwide exhibit deafness or various extents of hearing impairment (Murray et al., 2019; Olusanya et al., 2019); approximately 10% of these people (34 million) are children (Murray et al., 2019). Although this number does not fully reflect the non-negligible threat imposed by hearing loss on human health, an independent report from the National Institute on Deafness and Other Communication Disorders of the United States revealed that the fight against deafness was urgent (Wass et al., 2019). In the USA, over 15% of all people currently exhibit hearing loss or have previously exhibited hearing loss (Moeller, 2000). Hearing loss is often age-associated; individuals over 60 years of age tend to have hearing impairments (Uchida et al., 2019). However, deafness or hearing loss is even more serious in children, because hearing is closely related to language-learning, social behavior, and basic emotional development (Trudeau et al., 2021). Therefore, an exploration of the pathological factors associated with childhood deafness is critical for child health and of considerable interest to researchers. The clinical pathogenesis of hearing loss in children is either congenital (Korver et al., 2017) or acquired (Pichichero, 2018). Congenital causes have been associated with genetic factors and family histories (Korver et al., 2017). X-linked hearing loss is the most typical form of congenital hearing loss, passed from mothers to their sons (O’brien et al., 2021). Genes *PRPS1*, *POU3F4*, *SMPX*, *AIFM1*, and *COL4A6* have all been associated with X-linked hearing loss (Song et al., 2012). However, otitis media and ototoxicity also trigger childhood hearing loss (Vanneste and Page, 2019). Otitis media is a complex process that involves multiple infections and specific genetic susceptibilities (Vanneste and Page, 2019). Acute otitis media (the most common form of the condition) has been associated with infections by various bacteria including *Streptococcus pneumoniae*, *Hemophilus influenzae*, *Moraxella catarrhalis*, and *Staphylococcus aureus* (Deniz et al., 2018)*.* Additionally, acute otitis media susceptibility and recurrence have been associated with genetic factors. In 2011, researchers in Helsinki University Central Hospital reported that genetic factors contributed to childhood recurrent acute otitis media in 38.5% of affected patients and chronic otitis media in 22.1% of affected patients, highlighting the substantial contributions of genetic traits to these conditions (Hafrén et al., 2012). Furthermore, genome-wide association studies have shown that particular genes, including *FNDC1*, are associated with otitis media (Van Ingen et al., 2016), validating the essential roles of genetics in otitis media-induced hearing loss. Notably, drug ototoxicity was not significantly associated with the genetic background (Lanvers-Kaminsky et al., 2017). In summary, both congenital deafness and environmental otitis media (i.e., the two major pathogeneses of childhood hearing loss) feature strong genetic predispositions.

Although major efforts have been made to describe the pathogenesis of childhood hearing loss, the underlying mechanism remains unclear; only a few genes are known or suspected to be associated with the disease. Here, we focused on congenital hearing loss and otitis media-related hearing loss; both are associated with clear genetic predispositions. We used DisGeNet (https://www.disgenet.org/) to generate a list of genes associated with hearing loss (Piñero et al., 2017); we then employed a network-based method to identify novel latent biomarkers and genetic traits predisposing to congenital and otitis media-associated hearing loss. We used a random walk with restart (RWR) algorithm (Kohler et al., 2008; Macropol et al., 2009) by setting genes associated with otitis media or congenital deafness as the seed nodes to a STRING [19] protein-protein interaction (PPI) network to discover new candidate genes. A following screening procedure was conducted to select essential candidates. Eighteen latent congenital genes and 87 otitis media-associated genes were identified; some were associated with either pathogenesis. These may serve as novel biomarkers for clinical deafness screening in children; they will help to identify the pathogenetic mechanisms involved.

## Materials and Methods

### Genes Associated with Hearing Loss in Children

We focused on genes associated with hearing loss in children. The American Speech-Language-Hearing Association (Alsarraf et al., 1998; Dhooge, 2003) defines such hearing loss in children as either acquired or associated with otitis media or congenital deafness. We downloaded the relevant genes from DisGeNet (Piñero et al., 2017) (https://www.disgenet.org/, version 7.0, accessed in April 2021). In total, 175 genes were associated with otitis media, while 72 were associated with congenital deafness and 2 were associated with acquired hearing loss; thus, we did not study acquired hearing loss. The genes associated with congenital deafness and otitis media are listed in Supplementary Tables S1, S2, respectively. We used a network-based method to identify novel candidate genes associated with either pathogenesis.

### Network-Based Identification of Novel Genes

PPIs are widely used to explore protein or gene-related problems. Several studies have reported that compared with non-interacting proteins, interacting proteins are more likely to have similar functions (Ng et al., 2010; Hu et al., 2011; Chen et al., 2016a; Cai et al., 2017; Zhao et al., 2019; Gao et al., 2021). Such interactions can be used to identify novel genes that are associated with known disease-related genes. We used the STRING database (https://www.string-db.org/, version 10.0) (Szklarczyk et al., 2015) to construct a PPI network; we then applied the powerful, network RWR algorithm (Kohler et al., 2008; Macropol et al., 2009) to discover novel candidate genes associated with otitis media or congenital deafness. Human PPI information collected in STRING is contained in “9606.protein.links.v10.txt.gz”. Each PPI features two proteins identified by their Ensembl IDs, as well as a confidence score indicating the PPI strength. Each score ranges from 1 to 999 and is derived by considering several types of PPIs. In fact, PPIs in STRING can not only indicate the interactions between proteins but also reflect functional associations of proteins. Thus, they can widely measure protein associations. We used the PPIs to build a network in which all 19,247 proteins served as nodes. Two nodes were considered adjacent if and only if they formed a PPI; thus, each edge was a PPI. We assigned a weight to each edge for indicating the strengths of the PPI, which was defined as the confidence score of the corresponding PPI. The network was termed *N*.

The RWR algorithm is powerful. It simulates a walker that commences at a node set and then randomly moves in the network. The start nodes are termed seed nodes. The walker delivers probabilities of seed nodes to all other nodes in the network. Given a network and *k* seed nodes, each seed node is assigned a probability of 1/*k*; the other nodes are assigned probabilities of zero. These probabilities form a vector termed *P*_0_. The vector is repeatedly updated as follows:

$$P_{t+1}=\left(1-r\right)A^{T}P_{t}+rP_{0},$$ (1)

where *A* is the column-wise, normalized adjacency matrix of the network and *r* is the restarting probability, which was set to 0.8 in this study. Updating stops when $P_{t+1}$ and $P_{t}$ are sufficiently close; closeness is given by ${\left\|P_{t+1}-P_{t}\right\|}_{L_{1}}<10^{-6}$ . $P_{t+1}$ is the required outcome of the algorithm. Based on this outcome, each node is assigned a probability transmitted from the seed nodes. A higher node probability is indicative of stronger associations with seed nodes.

We used the RWR program established by Li and Patra (Li and Patra, 2010). Genes associated with congenital deafness or otitis media were fed into the program, which ran on the PPI network *N*. Nodes with probabilities higher than 10^−5^ served as raw candidate genes for congenital deafness or otitis media.

### Screening Procedure

Some raw candidate genes associated with congenital deafness or otitis media can be identified using a network-based method. However, several false-positives may be included in the results. To eliminate such genes and select only valid candidates, we used a screening procedure that featured three sequential tests.

### Permutation Test

The RWR algorithm was executed on the PPI network *N* to discover raw candidate genes. The structure of *N* may influence the outcome. Some nodes are readily assigned high probabilities because of their special locations in the network. However, they may have low or no associations with congenital deafness or otitis media. Thus, there is a need to test the statistical significance of the probability that each raw candidate gene is valid. Accordingly, we randomly generated 1,000 gene sets, each of which had the same number of genes associated with congenital deafness or otitis media. For each gene set, such genes were set as the seed nodes of the RWR algorithm. Thus, each candidate gene was assigned a probability in each random gene set. When all 1,000 sets had been tested, each candidate gene had been assigned 1,000 probabilities. By comparing the probability on actual seed nodes to the probabilities on randomly generated sets, the statistical significance of each probability was revealed. We used the Z-score to evaluate significance as follows:

$$Z-score\left(g\right)=\frac{P\left(g\right)-PM\left(g\right)}{PSTD\left(g\right)},$$ (2)

where *g* is a raw candidate gene identified by the network-based method, P(g) is the probability on actual seed nodes, and PM(g) and PSTD(g) are the respective mean and standard deviation of the probabilities on randomly produced sets. We set the selection threshold for candidate genes to 1.96; this is a widely accepted threshold when statistical significance is essential.

### Association Test

The second test directly evaluated the associations between candidate genes and congenital deafness or otitis media. For each candidate gene, such associations can be measured by associations between that gene and other genes associated with either condition. Proteins that interact in STRING always exhibit strong associations that can be quantified using confidence scores. For proteins *p* and *q*, the confidence score is denoted as Q(p,q) . For each candidate gene *g*, we computed the maximum association score (MAS) as follows:

MAS(g)=Max{Q(g,g′):g′ is a gene associated with congenital deafness or otitis media} (3)

Genes with high MAS values are strongly associated with at least one gene linked to congenital deafness or otitis media. Thus, such genes may also be highly related to either condition. We set the threshold for selection of essential candidate genes to 900; this is the cutoff of the highest STRING confidence score.

### Function Test

The last test further filtered candidate genes according to the similarities between their functional terms and the functional terms of genes associated with congenital deafness or otitis media. If the functional terms of a candidate gene are similar to the functional terms of a gene that is validly associated with either condition, that gene may also be linked to one of the conditions. We first used enrichment theory (Carmona-Saez et al., 2007; Huang et al., 2011; Huang et al., 2012; Chen et al., 2016b; Chen et al., 2019) to evaluate the associations between genes and functional terms (GO terms and KEGG pathway terms). Given one gene and one functional term, the gene set containing that gene and genes with which it interacted (in the PPI network of STRING) was constructed; another gene set containing genes annotated by the functional term was built. The associations between the gene and the functional term were calculated as the −log_10_ of the hypergeometric test *p-*value of the gene sets constructed above. For any gene *g*, its associations with all functional terms were computed and collected in a vector denoted V(g) . The similarity of two genes g and g' (based on their functional terms) can be evaluated by comparing their vectors as follows:

$$\Lambda \left(g,g\prime \right)=\frac{V\left(g\right)\cdot V\left(g\prime \right)}{\left\|V\left(g\right)\right\|\cdot \left\|V\left(g\prime \right)\right\|}$$ (4)

In a manner similar to MAS calculation, for each candidate gene *g*, the maximum function score (MFS) was computed as follows:

MFS(g)=Max{Λ(g,g′):g′ is a gene associated with congenital deafness or otitis media} (5)

Essential genes can be selected by choosing an appropriate MAS threshold.

### Functional Enrichment Analyses on Identified Genes

To explore biological functions associated with identified genes, we applied gene ontology (GO) enrichment analyses using R package *topGO* (https://bioconductor.org/packages/release/bioc/html/topGO.html, *v.2.42.0*). The threshold of *p*-value was set to 0.001 for selecting enriched GO terms in three subclasses: biological processes (BP), cellular components (CC) and molecular functions (MF).

## Results

**FIGURE 1:**
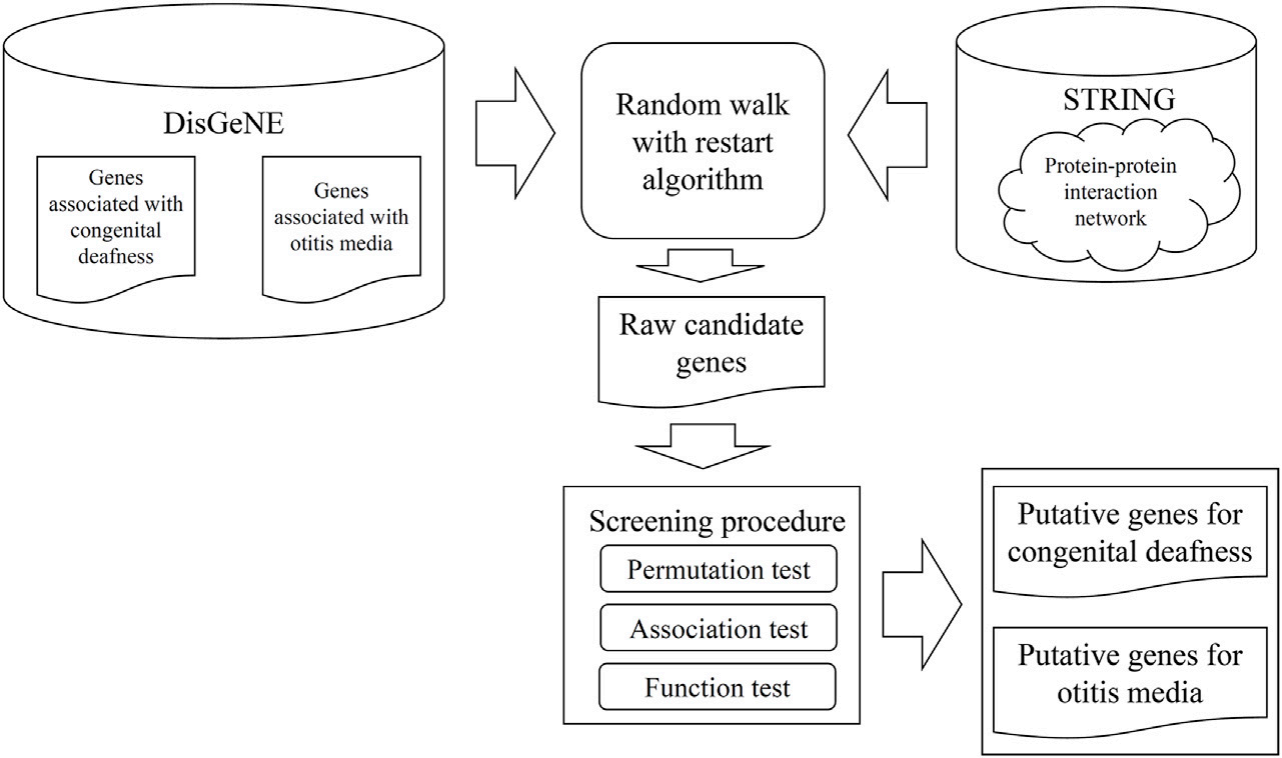
Procedures used to identify new genes that might have roles in the development of childhood congenital deafness or otitis media-mediated hearing loss. Genes associated with either pathogenesis were retrieved from DisGeNE and the STRING protein-protein interaction networks were explored. The genes and networks were fed into a random walk with restart algorithm; we sought to discover new candidate genes. These genes were screened using three tests to select putative genes.

We sought genes associated with pediatric congenital deafness or otitis media. We used a network-based method to identify such genes. The entire procedure is illustrated in Figure 1. The numbers of genes remaining after each filtration step are listed in Table 1.

**TABLE 1:** Numbers of candidate genes remaining after each filtration step.

Cause of childhood deafness	RWR	Permutation test	Association test	Function test
Congenital	5,426	367	117	18
Otitis media	5,631	637	502	87

### Congenital Deafness

Genes associated with congenital deafness were fed into the RWR algorithm, which ran on PPI network *N*. Each node in the network was assigned a probability. The selection threshold for raw candidate genes was set to 10^−5^; this yielded 5,426 genes (Supplementary Table S3). We then engaged in screening (i.e., filtration) to identify essential genes. First, we used the permutation test to evaluate the statistical significance of probability that each raw candidate gene was essential; the Z-scores for all genes are listed in Supplementary Table S3. In total, 367 candidate genes were assigned Z-scores greater than 1.96. These were fed into the association test, which assigned an MAS to each gene (Supplementary Table S3). At a threshold of 900, 117 genes were selected; these were finally evaluated using the function test. The MFS values are listed in Supplementary Table S3. At an MFS threshold of 0.9, 18 genes were chosen. These “putative genes” were considered to be closely associated with congenital deafness; they are listed in Supplementary Table S4.

**FIGURE 2:**
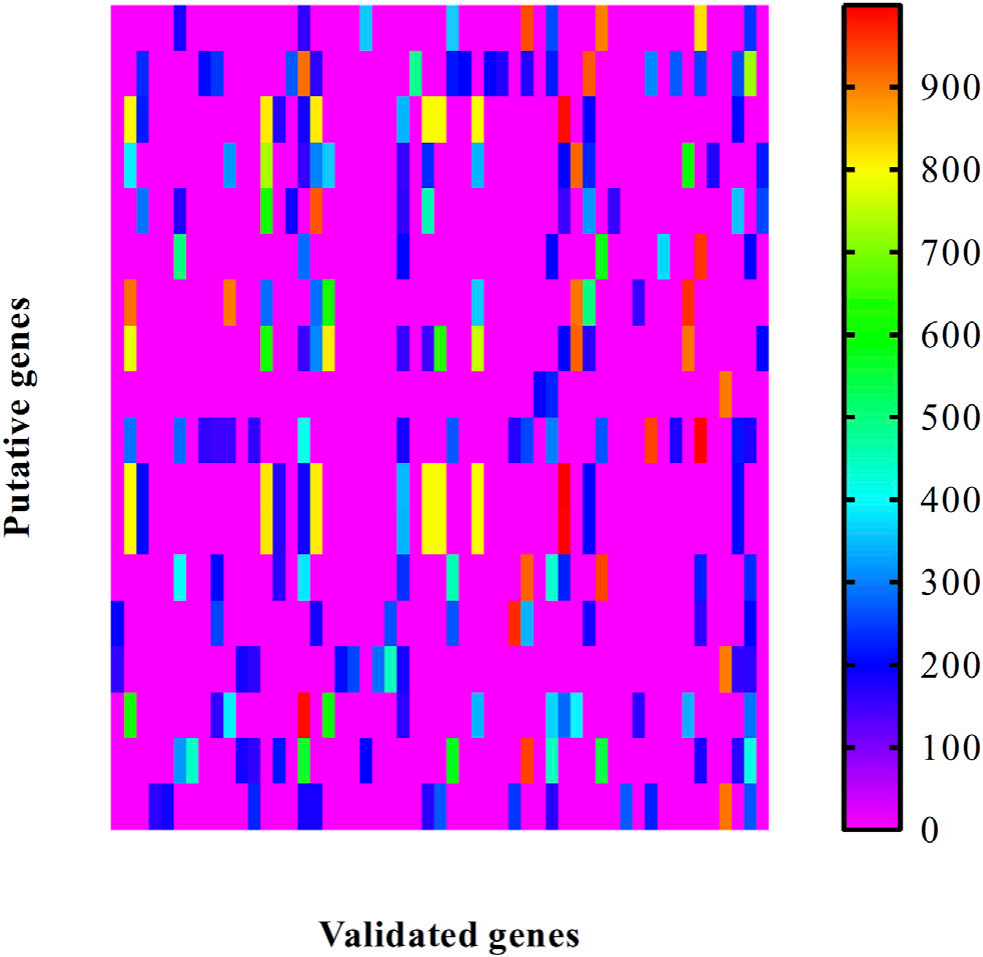
Heat map to illustrate the associations between putative genes and validated ones associated with congenital deafness. Row represents putative genes and column indicates validated genes.

For the obtained putative genes, their associations with validated genes were investigated. We extracted all PPIs between putative and validated genes. The confidence scores of these PPIs are illustrated in a heat map, as shown in Figure 2. It can be observed that each putative gene had some interacting genes with confidence scores no less than 900, suggesting strong associations with validated genes. This can be further inferred that putative genes had special relationships with congenital deafness.

### Otitis Media

We used the method described above to identify putative otitis media-associated genes. The RWR algorithm with genes associated with otitis media as seed nodes was performed on the PPI network *N*. The probabilities of all nodes were obtained. We selected nodes with probabilities over 10^−5^; this yielded 5,631 genes (Supplementary Table S5). These genes were filtered as described above. The Z-scores, MAS values, and MFS values are listed in Supplementary Table S5. Use of thresholds of 1.96 for the Z-score, 900 for the MAS, and 0.96 for the MFS yielded 87 “putative genes” (Supplementary Table S6).

**FIGURE 3:**
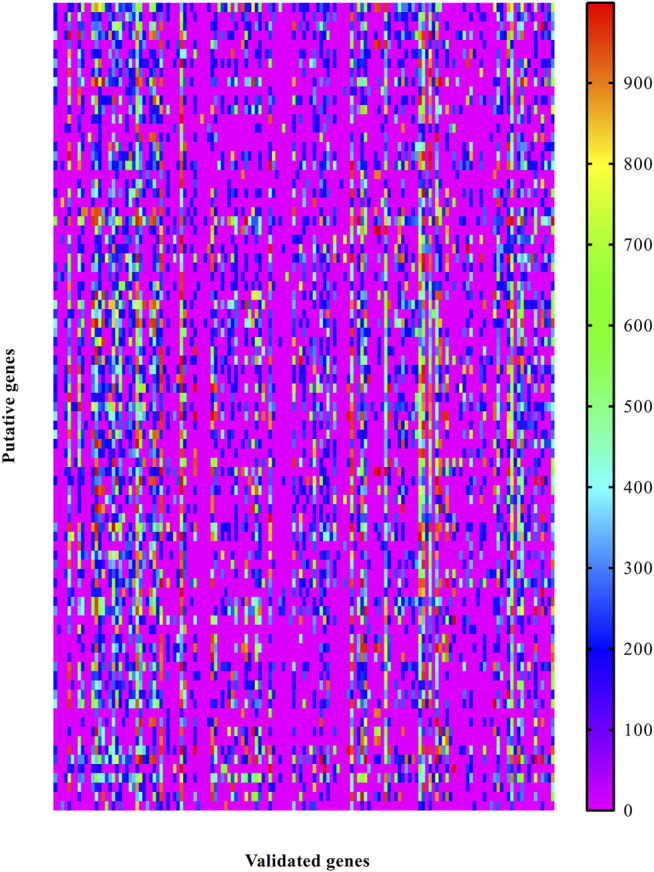
Heat map to illustrate the associations between putative genes and validated ones associated with otitis media. Row represents putative genes and column indicates validated genes.

Likewise, the PPIs between putative and validated genes were investigated. A heat map was plotted to indicate the strength of these PPIs, as shown in Figure 3. Also, each putative genes had one or more interacting genes with highest confidence (confidence score ≥900). It is suggested that these putative genes may have special associations with otitis media.

### GO Enrichment Analyses on Putative Genes

#### GO Enrichment Analyses on Congenital Deafness Associated Putative Genes

**FIGURE 4:**
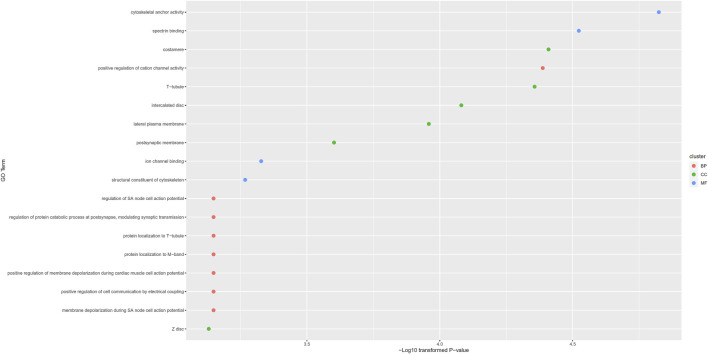
Gene ontology (GO) enrichment results for putative genes associated with congenital deafness. GO terms with *p*-value less than 0.001 are selected and ranked by their *p*-values.

For congenital deafness, 18 putative genes were obtained. These genes were set as gene of interest and all available genes were set as background for *topGO*. 18 enriched GO terms were obtained, which are provided in Supplementary Table S7. These terms and their *p*-values are also illustrated in Figure 4. Among these GO terms, eight were BP GO terms, six were CC GO terms and four were MF GO terms.

#### GO Enrichment Analyses on Otitis Media Associated Putative Genes

**FIGURE 5:**
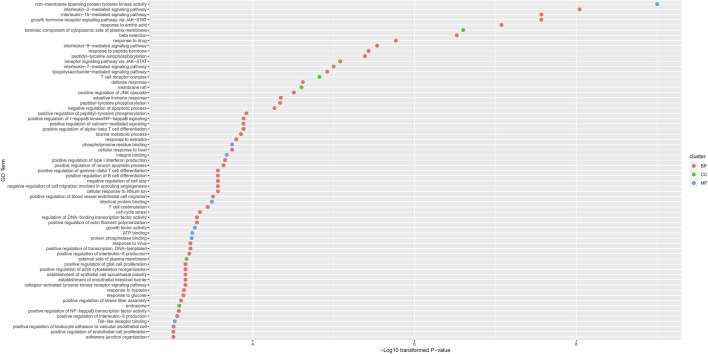
Gene ontology (GO) enrichment results for putative genes associated with otitis media. GO terms with *p*-value less than 0.001 are selected and ranked by their *p*-values.

For 87 putative genes associated with otitis media, we did the same enrichment analysis. Results are available in Supplementary Table S8. We obtained 65 enriched GO terms. These GO terms and their *p*-values are shown in Figure 5. Of these 65 GO terms, fifty-two belonged to BP, five belonged to CC and eight belonged to MF.

## Discussion

We used a network-based method to identify putative genes associated with congenital deafness or otitis media. Below, we discuss some genes.

### Putative Genes Associated with Congenital Deafness

We identified 18 putative genes, of which 5 were chosen for detailed analysis (Table 2). The first is ***PRKACB*** (ENSP00000359719), which encodes a catalytic subunit of cAMP-dependent protein kinase. The enzyme is expressed in hearing-associated organs *in utero*. In 2017, researchers from Southeast University showed that mouse *PRKACB* regulated the development of Lgr5+ hair cells (inner ear progenitor cells) (Cheng et al., 2017). Therefore, *PRKACB* is functionally associated with cochlear development; the cochlea is a sensorineural hearing organ. Cochlear impairment and abnormalities are reportedly associated with congenital hearing loss in children (O’malley et al., 1995; Korver et al., 2017; Van Wieringen et al., 2019). It is thus reasonable to expect that a regulator of cochlear development, such as *PRKACB*, would be associated with congenial pediatric deafness. We identified another putative gene with a similar biological function. ***PRKACG*** (ENSP00000366488) encodes another protein of the same complex. In 2016, researchers from the University of Bristol confirmed that the gain-of-function variant *DIAPH1* caused macrothrombocytopenia and hearing loss (Stritt et al., 2016). *PRKACG* acts downstream of *DIAPH1*, thus participating in *DIAPH1-*related biological effects. *PRKACG* may also be functionally connected to pediatric hearing loss.

**TABLE 2:** Five putative congenital deafness genes.

Ensembl ID	Gene symbol	Description	Probability	Z-score	MAS	MFS	Supporting References
ENSP00000359719	PRKACB	Protein Kinase CAMP-Activated Catalytic Subunit Beta	1.036E-04	2.2076	999	0.9884	O’malley et al. (1995), Cheng et al. (2017), Korver et al. (2017), Van Wieringen et al. (2019)
ENSP00000396259	PAX2	Paired Box 2	1.133E-04	5.0805	947	0.9877	Adam et al. (1993), Ziman et al. (2001), Zou et al. (2006), Kimura et al. (2018)
ENSP00000262848	PRKX	Protein Kinase X-Linked	1.018E-04	2.1608	987	0.9876	Song et al. (2012), Haltrich, (2019)
ENSP00000366488	PRKACG	Protein Kinase CAMP-Activated Catalytic Subunit Gamma	1.035E-04	2.1305	994	0.9862	Stritt et al. (2016)
ENSP00000378485	MATK	Megakaryocyte-Associated Tyrosine Kinase	6.746E-05	4.3589	986	0.9847	Jhun et al. (1995), Grgurevich et al. (1997), Lee et al. (2006), Costello et al. (2017)

The next putative gene is ***PAX2*** (ENSP00000396259), which is regarded as a key transcription factor that regulates the development of multiple systems, including the central nervous system (Ziman et al., 2001) and the eyes (Adam et al., 1993). In 2006, researchers from the McLaughlin Research Institute for Biomedical Sciences reported that *PAX2* interacted with *EYA1* to regulate the development of sensory regions in the inner ear (Zou et al., 2006). Developmental abnormalities of these regions are directly associated with congenital hearing loss (Kimura et al., 2018), implying that *PAX2* is a relevant putative gene involved in congenital pediatric deafness.

***PRKX*** (ENSP00000262848) is also associated with congenital pediatric hearing loss. A 2019 review concerning chromosomal aberrations associated with endocrine abnormalities in children confirmed that *PRKX* regulated the development of hearing (Haltrich, 2019). *PRKX* is located on the X chromosome; it is functionally connected to X-linked congenital hearing loss (Song et al., 2012).

The next putative gene is ***MATK*** (ENSP00000378485); this regulates signal transduction in hematopoietic cells (Grgurevich et al., 1997; Lee et al., 2006). In 2017, a clinical case report in *JAMA Otolaryngology—Head and Neck Surgery* stated that *MATK* was associated with unilateral hearing loss and otorrhea (Costello et al., 2017). Acute megakaryoblastic leukemia has been functionally connected to unilateral, congenital hearing loss; the pathogenetic backgrounds are related (Costello et al., 2017). MATK encodes megakaryocyte-associated tyrosine kinase, which is structurally similar to C-terminal Src kinase; notably, megakaryocyte-associated tyrosine kinase is associated with acute megakaryoblastic leukemia (Jhun et al., 1995). Therefore, *MATK* might be involved in the development of ear tumors that cause adaptive hearing loss.

### Putative Genes Associated with Otitis Media

We identified 87 genes putatively associated with otitis media (Supplementary Table S6); we subjected 5 of these genes to detailed analysis (Table 3 **)**. The first such gene is ***RAC3*** (ENSP00000304283). Although there is insufficient direct evidence that *RAC3* is involved in otitis media, a clinical genomic database (ClinVar Miner) (Henrie et al., 2018) indicates that the Talkowski Laboratory of Massachusetts General Hospital has demonstrated associations of *RAC3* variants with otitis media. The next gene is ***HCK*** (ENSP00000365012); this member of the Src tyrosine kinase family regulates the innate immune response (Ernst et al., 2002). *HCK* was previously reported to be specifically associated with chronic otitis media and its major chronic complications in children with hearing loss (Suri et al., 2016), validating our findings. The next putative gene is ***ITK*** (ENSP00000398655), which encodes an IL2-and T cell-associated kinase. In 2008, the gene was reported to potentially mediate the inflammation of otitis media (Juhn et al., 2008). Furthermore, a report concerning early diagnosis of PI3Kδ syndrome in a 2-year-old girl revealed an association between *ITK* deficiency and recurrent otitis media (Saettini et al., 2017).

**TABLE 3:** Five putative otitis media genes.

Ensembl ID	Gene symbol	Description	Probability	Z-score	MAS	MFS	Supporting References
ENSP00000304283	RAC3	Rac Family Small GTPase 3	1.496E-04	5.0315	994	0.9984	Henrie et al. (2018)
ENSP00000365012	HCK	HCK Proto-Oncogene, Src Family Tyrosine Kinase	6.876E-05	3.8668	985	0.9959	Ernst et al. (2002) Suri et al., 2016)
ENSP00000398655	ITK	IL2 Inducible T Cell Kinase	6.657E-05	4.7676	925	0.9959	Juhn et al. (2008), Saettini et al. (2017)
ENSP00000363115	FGR	FGR Proto-Oncogene, Src Family Tyrosine Kinase	5.308E-05	2.3092	955	0.9947	Klein et al. (1988), Kim et al. (2008), Vogelnik and Matos, (2017)
ENSP00000314458	CDC42	Cell Division Cycle 42	1.377E-04	2.2400	999	0.9946	Hoppe and Swanson, (2004), Kashani et al. (2021)

***FGR*** (ENSP00000363115; also known as *SRC2*), another member of the Src tyrosine kinase family, is also associated with otitis media. This gene has roles in immune responses against pathogens in multiple organs, including ears (Kim et al., 2008). Additionally, the gene has been widely reported to participate in Epstein–Barr virus-associated malignancies (Klein et al., 1988). In 2017, Epstein–Barr virus infection was confirmed as a major etiological and pathological factor for secretory otitis media in children (Vogelnik and Matos, 2017), validating the link between *FGR* and otitis media.

***CDC42*** (ENSP00000314458) is an immune system-associated gene; we found that it was closely associated with otitis media. In 2021, *CDC42* deficiency was shown to be associated with recurrent pneumonia, otitis media, and bacteremia (Kashani et al., 2021). *CDC42* interacts with another effector gene, *RAC1* (Hoppe and Swanson, 2004); a homolog of *RAC1* (i.e., *RAC3*, discussed above) was shown to be associated with hearing loss, confirming that *CDC42* is linked to otitis media.

In summary, several putative genes are associated with the two types of pediatric deafness. Their identification may provide insights concerning the pathogeneses involved.

### Functional Enrichment Analyses on Putative Genes

For GO functional enrichment analyses on putative genes yielded by our computational method, multiple significant GO terms were identified. The detailed analyses on the top three enriched GO terms ranking by p-values for congenital deafness and otitis media were presented below.

For congenital deafness, the first enriched GO term is cytoskeletal anchor activity (GO:0008093). According to recent publications, mutations in cytoskeletal encoding proteins have been shown to be associated with congenital deafness (Riazuddin et al., 2006), reflecting the potential associations between congenital deafness and cytoskeletal anchor activity. The second enriched term is spectrin binding (GO:0030507). In 2017, a recessive mutation on spectrin associated gene has been shown to be associated with congenital central deafness (Knierim et al., 2017), validating this result. Furthermore, costamere (GO:0043034) is the third enriched GO term (in CC) associated with congenital deafness. According to recent next-generation sequencing analyses (Schraders et al., 2011), costameres has been shown to be associated with progressive hearing impairment.

More GO terms were enriched by putative genes associated with otitis media, including non−membrane spanning protein tyrosine kinase activity (GO:0004715) and interleukin mediated signaling pathway (GO:0038100, GO0035723). Non-membrane spanning protein tyrosine kinase has been shown to be associated with specific inflammatory effects and pathogen infections (Gu et al., 2009; Rocha-Sanchez et al., 2013). Considering that otitis media is associated with infection and inflammatory effects around middle ears, it is reasonable for otitis media associated genes to enrich in inflammatory effects. As for interleukin mediated signaling pathways, middle ear inflammation has been shown to be associated with interleukin related signaling pathways, validating this result (Kerschner et al., 2006; Shi et al., 2014).

### Shared Putative Genes Associated with Both Congenital Deafness and Otitis Media

By comparing the putative genes associated with congenital deafness and otitis media, only one shared gene ***CDH1*** (ENSG00000039068) was identified. The pathogenesis of congenital deafness and otitis media are totally different according to recent studies. Congenital deafness means the hearing loss is present at birth linking the pathogenesis to genetic factors or stimulations during pregnancy. However, as for otitis media, generally, otitis media is caused by infections and happens after birth. *CDH1* has been widely reported to be associated with hearing loss (Friedman and Avraham, 2009; Kanavy et al., 2019). Specifically, *CDH1* has been reported to be associated with congenital deafness due to the pathogenic alteration of inner ear but not middle ear (Friedman and Avraham, 2009), which has totally different pathogenic regions comparing with otitis media. As for otitis media, *CDH1* has been shown to participate in the pathogenesis of otitis media *via* regulation on the inflammatory proliferative responses against infections (Kurabi et al., 2013).Therefore, although both subtypes of hearing loss have been shown to be associated with gene *CDH1*, the contribution and regulatory role of *CDH1* on them are totally different, reflecting the complex regulatory mechanisms for childhood hearing loss.

In summary, *CHD1* is associated with two types of pediatric deafness. Its dentification may provide insights concerning the pathogeneses involved.

## Conclusion

We used a network-based method to identify new candidate genes involved in childhood hearing loss caused by congenital deafness and otitis media. The genes included *PRKACB*, *PAX2*, *PRKX*, *PRKACG*, *MATK, RAC3*, *HCK*, *ITK*, *FGR*, and *CDC42*. They may be involved in the pathogenesis of childhood hearing loss.

## Data Availability

Publicly available datasets were analyzed in this study. This data can be found here: https://www.disgenet.org/.
